# High-Strength β-Phase Magnesium–Lithium Alloy Prepared by Multidirectional Rolling

**DOI:** 10.3390/ma16083227

**Published:** 2023-04-19

**Authors:** Zhengyou Guo, Qing Ji, Ruizhi Wu, Haoyang Jia, Di An, Xiaochun Ma, Siyuan Jin, Jiarui Li, Jinyang Liu, Huajie Wu, Jinghuai Zhang, Legan Hou

**Affiliations:** 1 College of Materials Science and Chemical Engineering, Harbin Engineering University, Harbin 150001, China; 2 College of Materials Science and Engineering, Taiyuan University of Science and Technology, Taiyuan 030024, China

**Keywords:** bcc Mg–Li alloy, multidirectional rolling, rolling temperature, nanograins, dislocation

## Abstract

Magnesium–lithium alloys are popular in the lightweight application industry for their very low density. However, as the lithium content increases, the strength of the alloy is sacrificed. Improving the strength of β-phase Mg–Li alloys is urgently needed. The as-rolled Mg-16Li-4Zn-1Er alloy was multidirectionally rolled at various temperatures in comparison to conventional rolling. The results of the finite element simulations showed that multidirectional rolling, as opposed to conventional rolling, resulted in the alloy effectively absorbing the input stress, leading to reasonable management of stress distribution and metal flow. As a result, the alloy’s mechanical qualities were improved. By modifying the dynamic recrystallization and dislocation movement, both high-temperature (200 °C) and low-temperature (−196 °C) rolling greatly increased the strength of the alloy. During the multidirectional rolling process at −196 °C, a large number of nanograins with a diameter of 56 nm were produced and a strength of 331 MPa was obtained.

## 1. Introduction

Magnesium (Mg) alloys have emerged as one of the industry’s most popular options to accommodate the demand for lower weight. Mg alloys have excellent specific strength and stiffness because of their low density. J. Wang et al. studied the use of accumulative roll bonding (ARB) to strengthen the electromagnetic shielding performance of a duplex-phase Mg–9Li–3Al–1Zn alloy, which inspired the future design of lightweight and high-strength electromagnetic shielding structural materials [1] with excellent dampening and electromagnetic shielding properties [2,3]. Because of these advantages, Mg alloys offer a wide range of possible applications in aerospace, military equipment, medical equipment, 3C electronics, etc. They have a solid basal structure and are difficult to manufacture because of the close-packed hexagonal (hcp) structure of Mg [4,5]. The addition of Li can improve the plasticity of Mg alloys. The density of Li is 0.53 g/cm^3^, and it has a body-centered cubic (bcc) structure. Li further decreases the density of Mg alloys. Additionally, the crystal lattice’s c/a value is decreased; that is, it gradually evolves from the hcp structure to the bcc structure [6]. When plastic deformation occurs, the presence of the bcc structure increases the number of slip systems. Single-phase α-Mg (<5.7 wt.%), two-phase α-Mg + β-Li (5.7–10.3 wt.%), and single-phase β-Li (>10.3 wt.%) are the phases that correspond to varying Li contents in Mg alloys [7,8]. To obtain lightweight alloys and good ductility, 14 wt.% Li or more is frequently added to Mg alloys [9]. However, the strength of the Mg–Li alloy suffers sharply as the Li percentage rises [10,11].

It is common practice to use plastic deformation methods such as extrusion, rolling, and torsion to improve the strength of β-phase Mg–Li alloys [7,12]. For example, T. Mineta et al. effectively improved the hardness and corrosion resistance of LA143 alloy by accumulative channel-die compression bonding (ACCB) as a severe plastic deformation (SPD) technology [13]. The bcc structure had low stacking fault energy and a large dislocation range. Even at room temperature and under mild stress, the alloy experienced dynamic recrystallization (DRX) and dislocation plugging during the plastic deformation process [14,15]. As a result of the low critical shear stress, the dislocation plugging site was prematurely activated [4,16]. Therefore, the strength of the β-phase Mg–Li alloy was sensitive to temperature and deformation.

The aim of this research, based on the LZE1641 alloy processed by conventional rolling at room temperature, was to further increase the alloy’s strength using multidirectional (MD) rolling and altering the rolling temperature under the condition of the same equivalent strain. The alloy underwent MD rolling as opposed to conventional rolling. The microstructure and mechanical characteristics of the alloy were examined in different states.

## 2. Material and Methods

### 2.1. Alloy Preparation and Processing

**Figure 1:**
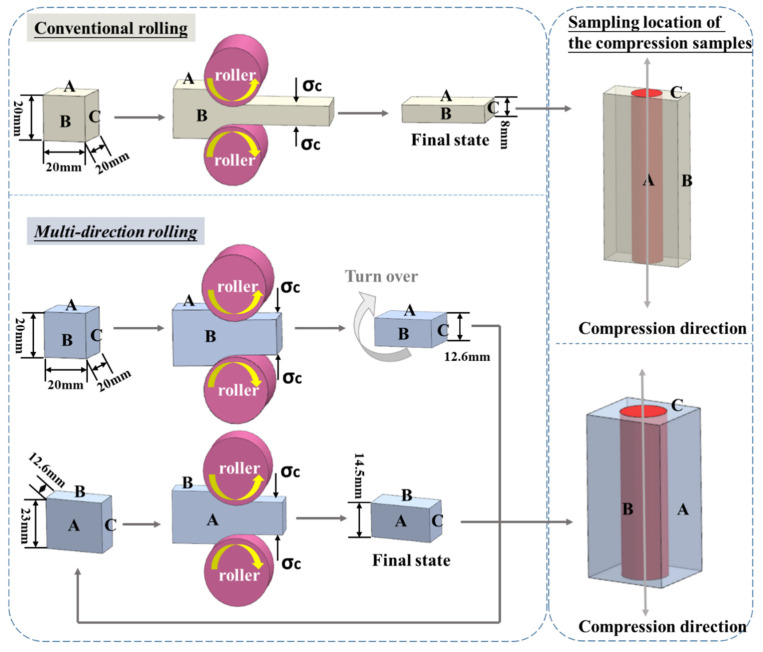
Schematic diagram of rolling and the locations of compression samples in as-rolled samples [18].

The composition of the alloy was Mg–16Li–4Zn–1Er (wt.%). The ingots for the alloy were Mg (>99.9 wt.%), Li (>99.9 wt.%), Zn (>99.9 wt.%), and Mg-20 wt.% Er. The ingots were melted in a medium-frequency vacuum induction furnace under the protection of an Ar environment. Then, the melt was poured into a permanent mold. The dimensions of the as-cast specimen were 120 × 110 × 40 mm^3^. The as-cast specimen was cut into 20 × 20 × 20 mm^3^ blocks for the ensuing rolling. The samples were subjected to conventional and MD rolling, respectively. The reduction of each pass was 0.8 mm. The specifics are displayed in Figure 1. The conventional rolling was decreased by 60%, implying that a single surface (A-side) was consistently maintained as an RD (rolling direction)–TD surface (transverse direction). The samples were deformed from the initial thickness of 20 mm to 8 mm. The alloy’s equivalent strain law during the rolling process is represented by the following formula [17]:

(1) $$\varepsilon =\frac{2}{\sqrt{3}}\left|\ln\left(\frac{h_{0}}{h}\right)\right|$$

The equivalent strain of MD rolling was the same as that of conventional rolling, $\varepsilon_{MDR}$ = $\varepsilon_{R60\%}$ = 1.05804. The distinction was that in MD rolling, two surfaces, A-side and B-side, alternately served as the RD–TD surface. The two surfaces bore half the corresponding strain, $\varepsilon_{A-MDR}$ = $\varepsilon_{B-MDR}$ = 1/2$\varepsilon_{MDR}$. The actual investigations revealed that when the samples were deformed, the width of the TD direction was difficult to keep at 20 mm, and it typically expanded to 23 mm. As a result, when the A-side was first used as the RD–TD surface, it experienced deformation from a thickness of 20 mm to 12.6 mm. Then, the sample was turned over and the B-side became the RD–TD surface, and the thickness was decreased from 23 mm to 14.5 mm.

The samples were rolled at three different temperatures: 25, 200, and −196 °C [15]. Specimens were placed in a vacuum heat treatment furnace for 20 min before rolling at 200 °C and kept in the furnace for 5 min between two rolling runs. Specimens were cryogenically rolled after being submerged in liquid nitrogen for 10 min before rolling and for 5 min between two rolling passes. In the end, alloys with six states were created, namely 25 °C rolled, 200 °C rolled, −196 °C rolled, 25 °C MD rolled, 200 °C MD rolled, and −196 °C MD rolled.

### 2.2. Microstructural Characterization

Scanning electron microscopy (SEM) and transmission electron microscopy (TEM) were used to analyze the specimens’ microstructures. The SEM and TEM equipment models were the FEI Talos F200X G2 (FEI, Brno, Czech Republic) and TEM-2100EX (JEOL, Tokyo, Japan), respectively. Wire electrical discharge machining was used to cut off samples with a thickness of 0.5 mm, and then the thin slices were polished with 3000# sandpaper to achieve a thickness of 45 μm. Finally, a wafer of ϕ3 mm was removed from each slice after ion thinning.

The average grain and second-phase diameters were determined using a mean linear intercept approach.

### 2.3. Compression Test Analysis

Because of the dimension limitations of rolled samples, particularly the MD rolled samples, tensile tests were challenging to conduct. We used a compression test to compare the strength of the six alloys. In this study, gleeble-3500 hot compression was used to test the compressive properties of the alloys. The size of the compression sample was ϕ8 mm × 12 mm. The location of the compression sample within the rolled sample is depicted in Figure 1. Each specimen was subjected to a strain rate of 1.0 × 10^−2^ s^−1^, and the engineering strain was 70%. Five compression samples were made and evaluated for alloy in each state and averaged as the final result.

## 3. Results

### 3.1. Mechanical Properties

**Figure 2:**
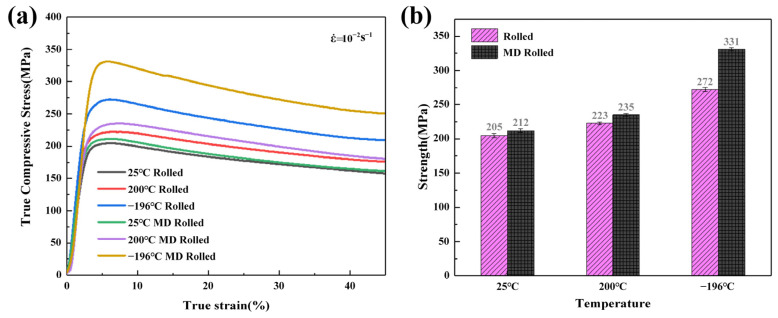
(**a**) Valid segment of compressive true strain-true stress curve; (**b**) histogram of compressive strength.

The valid portions of the compressive accurate strain-true stress curves are shown in Figure 2a. The yield strengths (*σ*_y_) and compressive strengths (*σ*_bc_) of the alloys are displayed as histograms in Figure 2b. As for conventional rolling, both elevated-temperature and cryogenic rolling could increase the strength of the alloy compared to rolling at normal temperature. Cryogenic rolling caused a more noticeable increase in strength. The strength fluctuation resulting from MD rolling at various temperatures was equivalent to that of conventional rolling. Compared to conventional rolling, MD rolling could further increase the strength; that is, compressive strength could reach up to 331 MPa.

### 3.2. Microstructures

**Figure 3:**
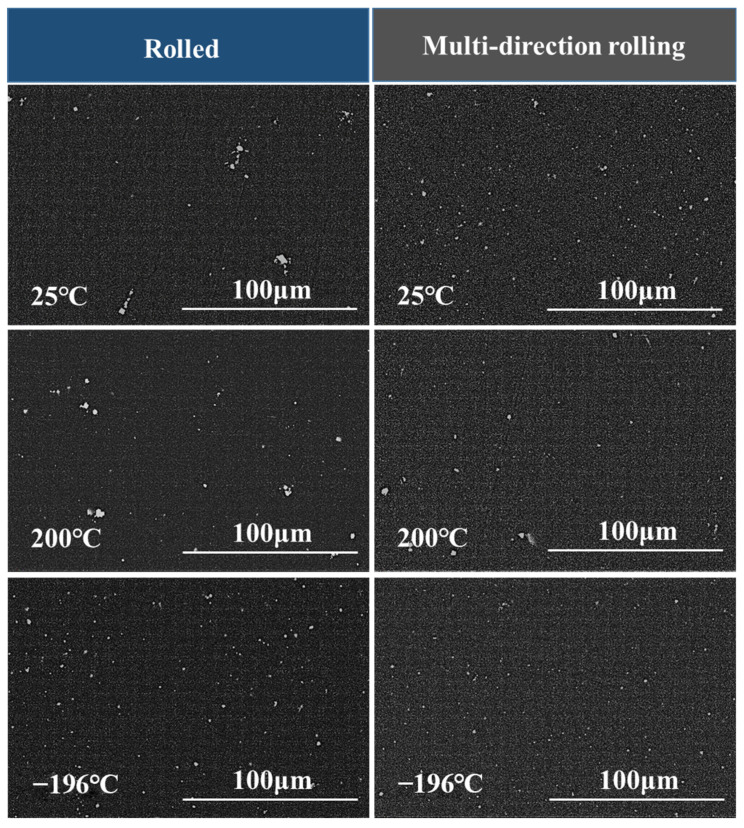
SEM images of the fragmentized primary second phase under various circumstances.

**Figure 4:**
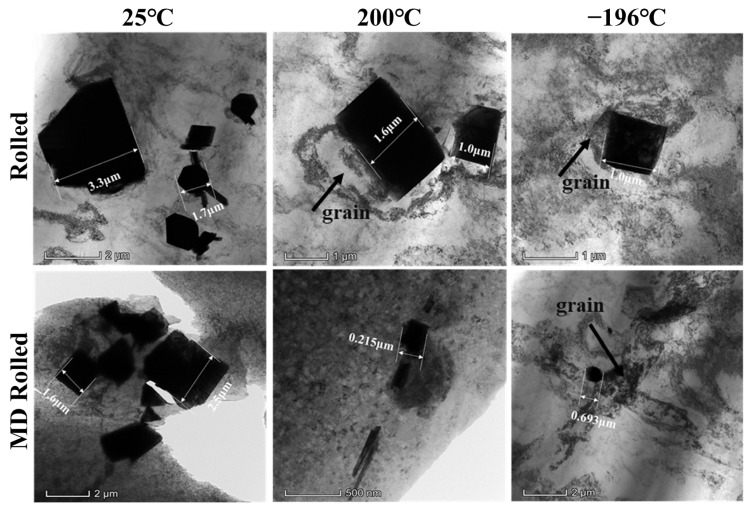
TEM images of the fragmentized primary second phase under various circumstances.

**Figure 5:**
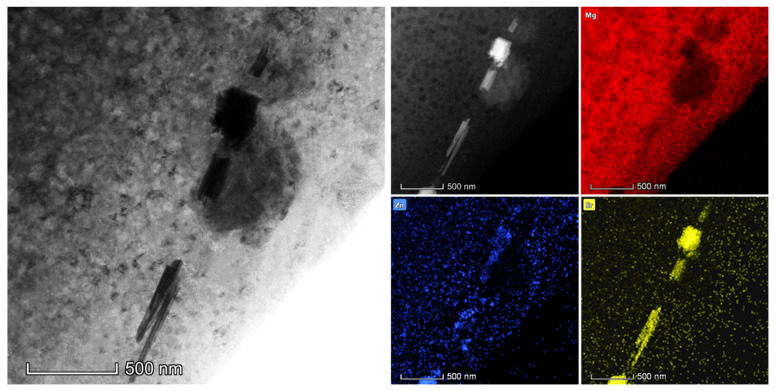
Mapping of the fragmentized primary second phase.

Among various deformation techniques, MD rolling can considerably improve the uniform distribution of primary second-phase fragments with an acceptable size in the matrix. According to the SEM results in Figure 3, the impact of elevated-temperature rolling on the crushing of the coarse second-phase was unclear under the same deformation procedure. However, cryogenic rolling could crush the coarse primary second phase and evenly distribute the fragments. This illustrated the uniformity of stress distribution during MD rolling. Figure 4 shows the TEM images for the broken second phase. The crushed fragments were spread and gathered around the original locations of the primary second phase, and they could not be effectively and evenly dispersed within the matrix. The size of the second-phase fragments was still quite substantial, exceeding micrometers. Meanwhile, the fragments were completely dissolved and there was no adhesion between the fragments in the −196 °C rolled alloy. In particular, −196 °C MD rolling had the greatest impact on lowering the size of the second-phase fragments and promoting the uniform distribution of fragments in the matrix, which was in line with the SEM data. Additionally, the grains leeched onto the boundaries of the primary second phase. The results proved that the primary second phase successfully underwent DRX. Figure 5 shows the element distribution of the primary second phase. According to the findings, Zn and Er made up the elemental composition of the particles. Furthermore, there were a considerable number of Er atoms with a dense distribution, indicating that Er > Zn. Additionally, Zn and Er components dissolved in the matrix to provide solid solution strengthening. This outcome was in line with earlier SEM and TEM findings.

**Figure 6:**
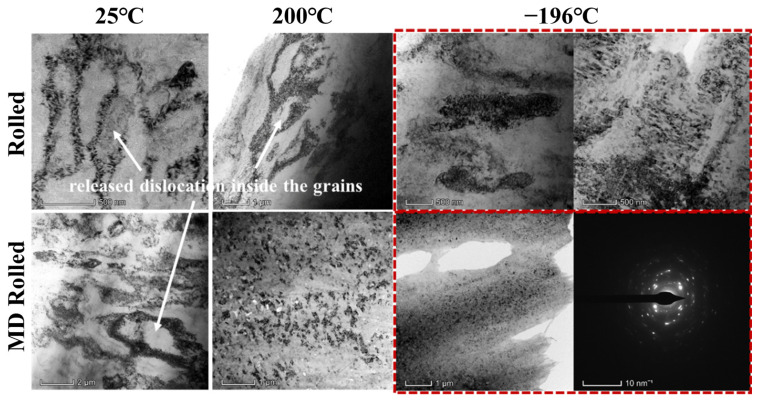
Bright-field TEM images of sub-grains under distinct circumstances.

In order to remove the interference of the primary second phase on the growth trend of grains and the movement behavior of dislocations, the areas without second-phase fragments were studied using bright-field TEM, as shown in Figure 6. The grains in the images were all DRX grains. After conventional rolling at 25 °C, the dislocations at grain borders were sparsely distributed and they were released inside the grains. Rolling at 200 °C resulted in the dislocations being more densely distributed toward the grain borders, but the dislocations inside the grains were still released or almost completely gone. Cryogenic rolling resulted in the grains being filled with densely distributed dislocations. Obviously, cryogenic therapy could postpone the release of dislocations within the grains. Additionally, some nanograins existed in the matrix with a size of 80~120 nm. On the other hand, MD rolling had a more obvious impact on dislocation buildup and grain refinement. The dislocation distribution at the grain boundaries of specimens MD rolled at 25 °C was narrower than that of the conventionally rolled specimens. When the rolling temperature rose to 200 °C, the grains transformed into nanoscale grains, the size of which was about 90 nm. When the alloy was MD rolled at −196 °C, a variety of uniformly distributed nanograins with a diameter of roughly 56 nm appeared. The diffraction spot images were all ring-shaped spots when collected in any area of the nanocrystal. According to the findings, the dislocations were not released inside the grains when the grain size was small enough.

## 4. Discussion

Based on the results presented above, the following relationships can be described:

(1) The compressive strength of the alloy is proportional to the grain size. When nanoscale grains form in an alloy, the compressive strength of the alloy dramatically increases.
(2) Whether or not the dislocations inside the grains are released does not depend on the temperature or the method of deformation but only depends on the grain size. When the grain size is below a micrometer-scale level, dislocations are prevented from being released and the alloy’s strength is quite high.

In conclusion, the DRX grains were responsible for the effects of both rolling temperature and direction on the microstructure. As a result, we split the discussion into two sections: (1) the control of rolling temperature on DRX grains and (2) the control of MD rolling on DRX grains.

### 4.1. The Control of Rolling Temperature on DRX Grains and Dislocations

The initial grain size of Mg–Li alloys is very large, and DRX is frequently seen during plastic deformation. As a result, grain refining is one of the most effective methods of improving strength. Controlling the nucleation and growth of DRX grains is particularly crucial. The DRX speed ($V_{DRX}$) conforms to the Arrhenius formula [19]:

(2) $$V_{DRX}=A\;\cdot \;e^{-Q_{R}/RT}$$

where *A* is the Arrhenius constant; $Q_{R}$ is the experimental activation energy, typically thought of as a constant independent of temperature; *T* is the absolute temperature; and *R* is the molar gas constant. It is evident that $V_{DRX}$ ∝ *T*; that is, elevated rolling temperature causes elevated DRX speed. In this study, the strengthening impact of 25 and 200 °C on the alloy mainly depended on the grain boundary growth brought on by DRX with the proliferation and migration of activated dislocations. The critical driving force needed to trigger DRX in β-phase Mg–Li alloys was low [20]. The external stress was sufficient at room- or elevated-temperature deformation to activate DRX.

#### 4.1.1. Elevated Temperature

High-temperature deformation leads to the storage of energy and is the driving force for the alloy to prematurely reach the critical value. The interface energy of DRX grains in the early process of nucleation is high, and the driving force of grain boundary movement is significant [21]. Driven by excess heat and stress input, it is simpler for the grains to multiply and engage in deformation, and even a second DRX occurs [22]. The primary distinction between the two temperatures was that 200 °C altered the microstructures more dramatically; meanwhile, the nucleation and growth rates of DRX were accelerated [23]. Rolling at 200 °C could speed up the propagation of dislocations in the matrix. The grain boundary still maintained the hindering effect on dislocation slip [24]. However, under elevated-temperature deformation, the dislocations moved more quickly, leading to an untimely blockage. This encouraged the occurrence of DRX even more. At the same time, dislocations were also released in significant numbers. When the dynamic equilibrium was destroyed, there were packed dislocations at the grain boundaries but no dislocations within the grains. Elevated-temperature rolling could not cause the nucleation rate of DRX to be more than the growth rate. Thus, there was no change in the DRX grain size of the alloy rolled at 200 °C compared with that rolled at 25 °C. The alloy was strengthened by rolling at 200 °C due to an increase in the number of dislocations.

#### 4.1.2. Cryogenic Temperature

Cryogenic rolling greatly slowed down DRX in the alloy [25]. External stress has been identified as the main driver of DRX nucleation and growth. The extremely low temperature considerably increased the pushing power of migration for DRX grain boundaries [26]. After the nucleation of DRX grains, there was not enough power to support grain boundary migration. This widened the distance between the nucleation and growth rates of DRX, resulting in the retention of more nanocrystals [27]. The rate of work hardening of deformed materials also largely depends on temperature. The work-hardening rate often rises with a decrease in temperature because of the energy-release process that takes place during or right after the deformation [28]. The non-uniform deformation strength during low-temperature deformation was significantly higher than that at room- and high-temperature deformation. This resulted in higher deformation energy storage. As a result, −196 °C rolling improved the strength of the alloy above the other two methods of fine grain strengthening and dislocation reinforcement.

### 4.2. The Control of MD Rolling on DRX Grains and Dislocations

**Figure 7:**
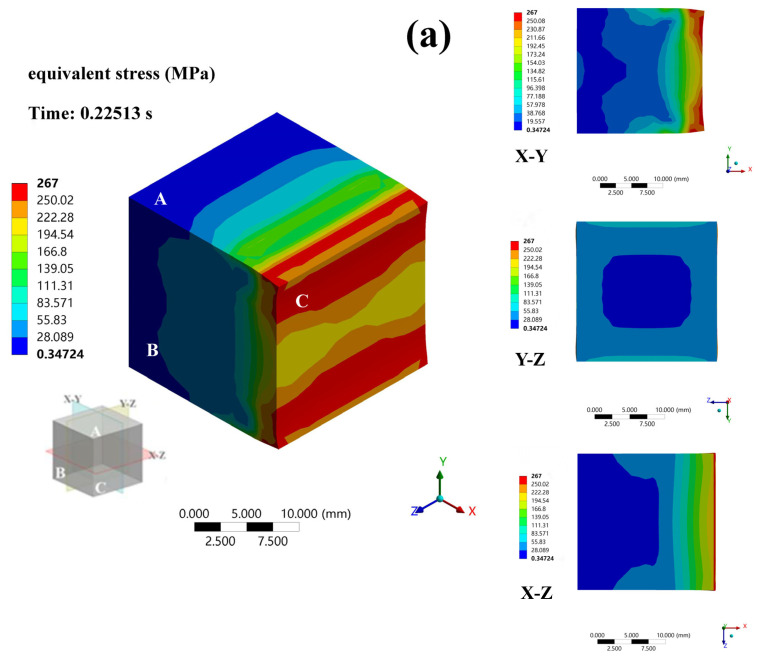

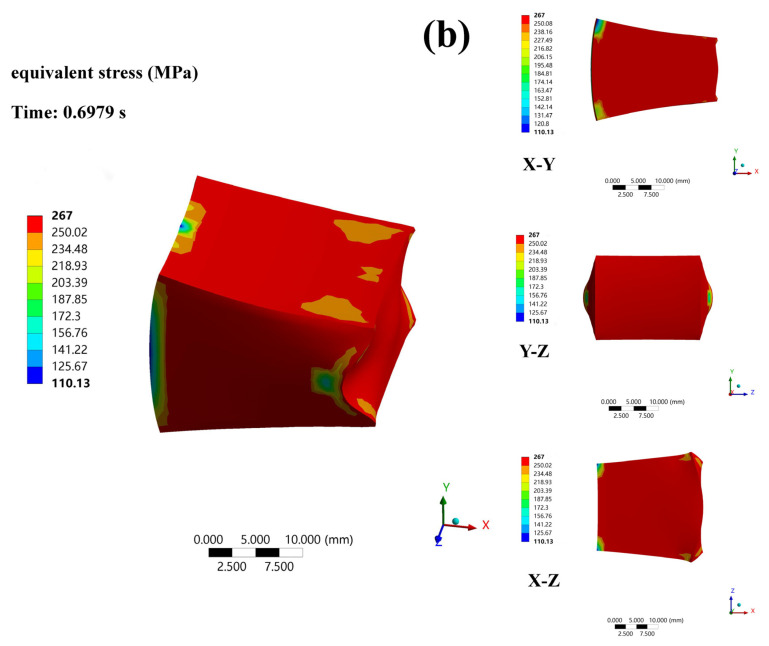

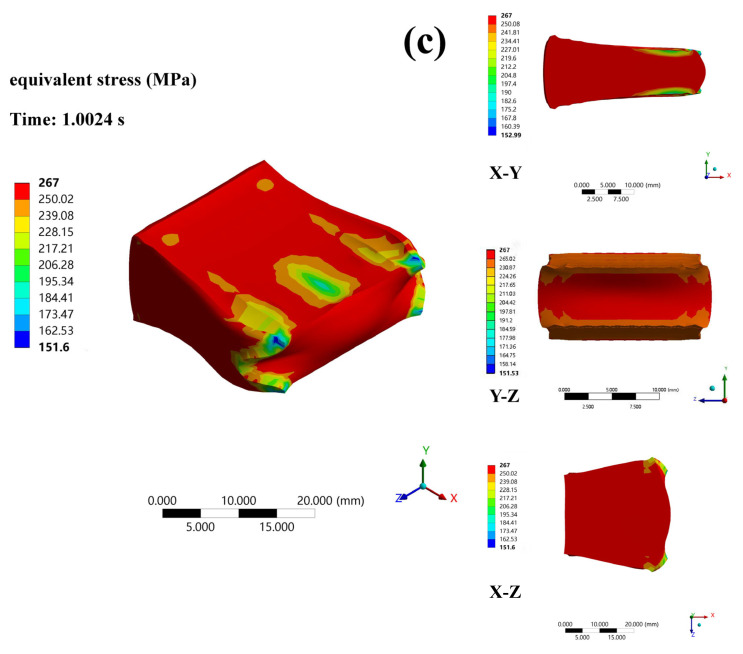

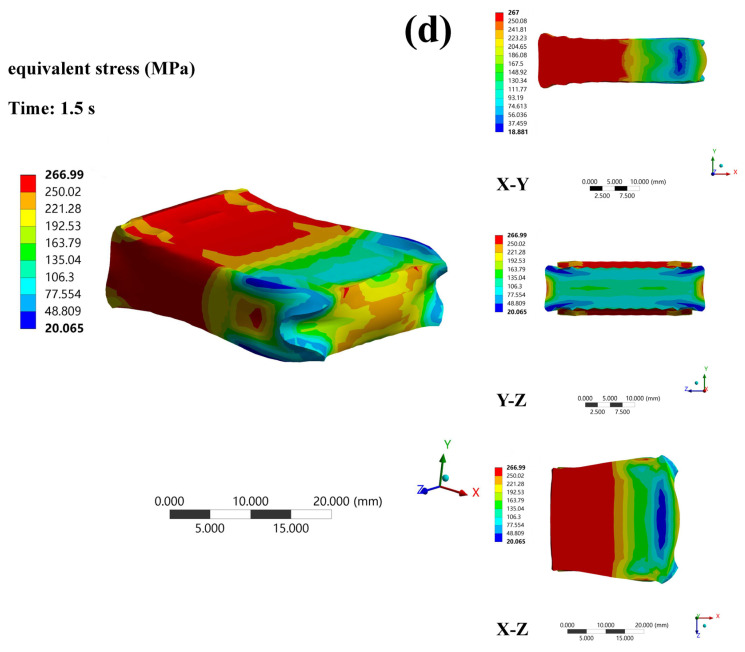

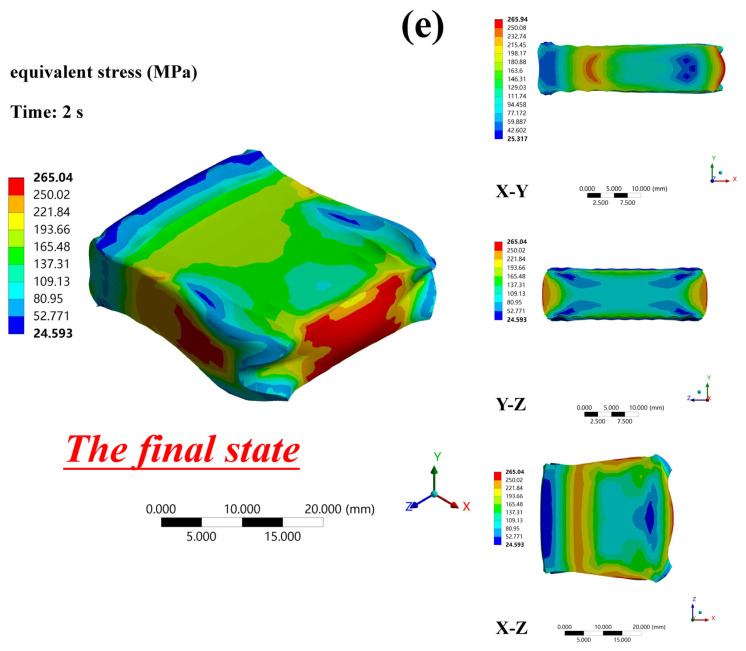
The equivalent stress distribution of conventional rolled alloy. (**a**) Deformation state of 0.22513 s; (**b**) Deformation state of 0.6979 s; (**c**) Deformation state of 1.0024 s; (**d**) Deformation state of 1.5 s; (**e**) Deformation state of 2.0 s.

**Figure 8:**
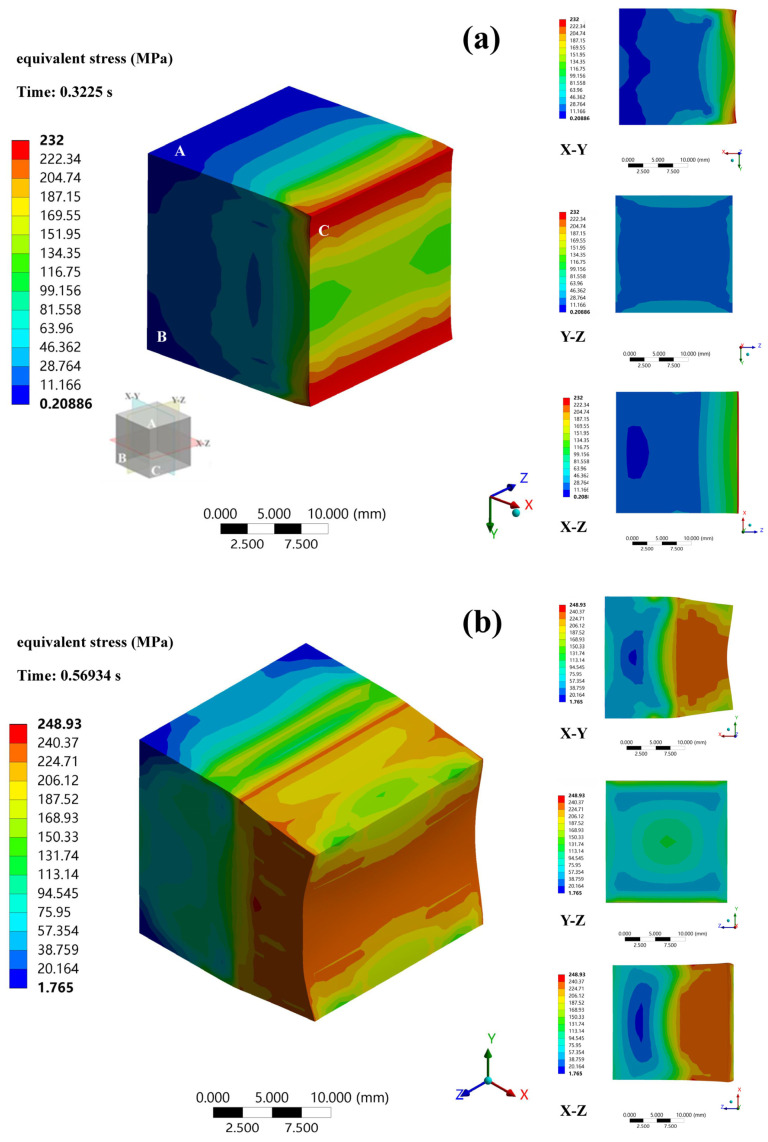

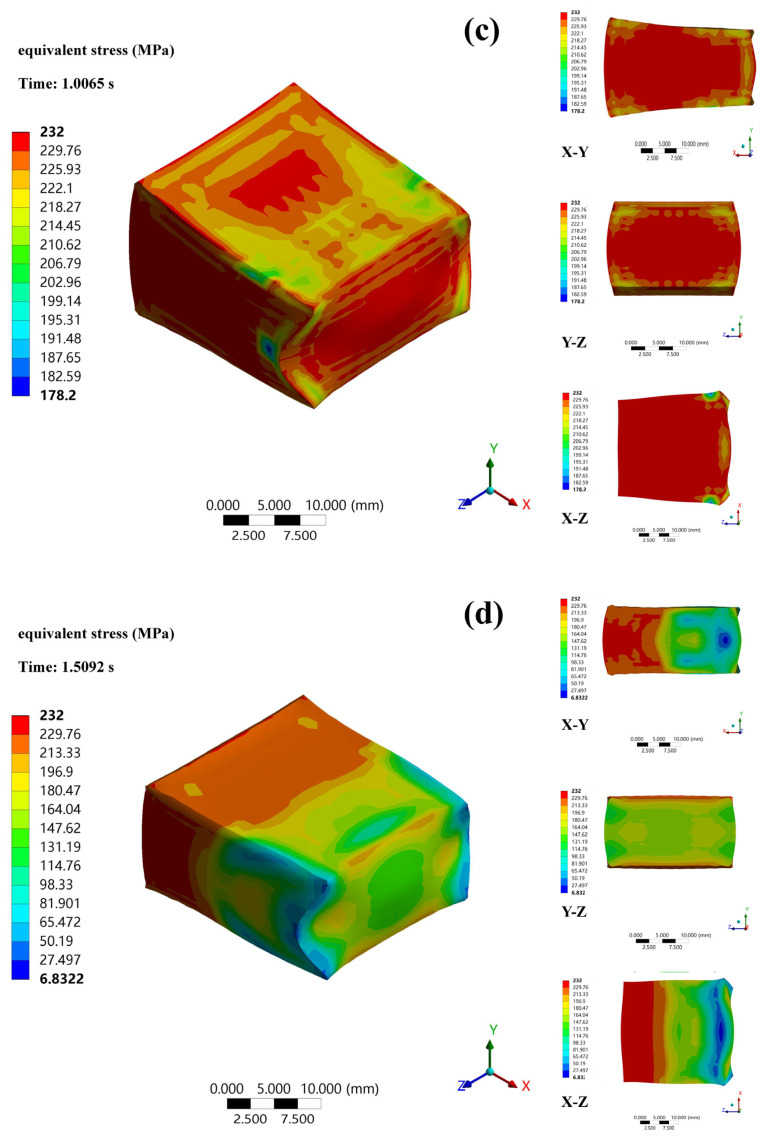

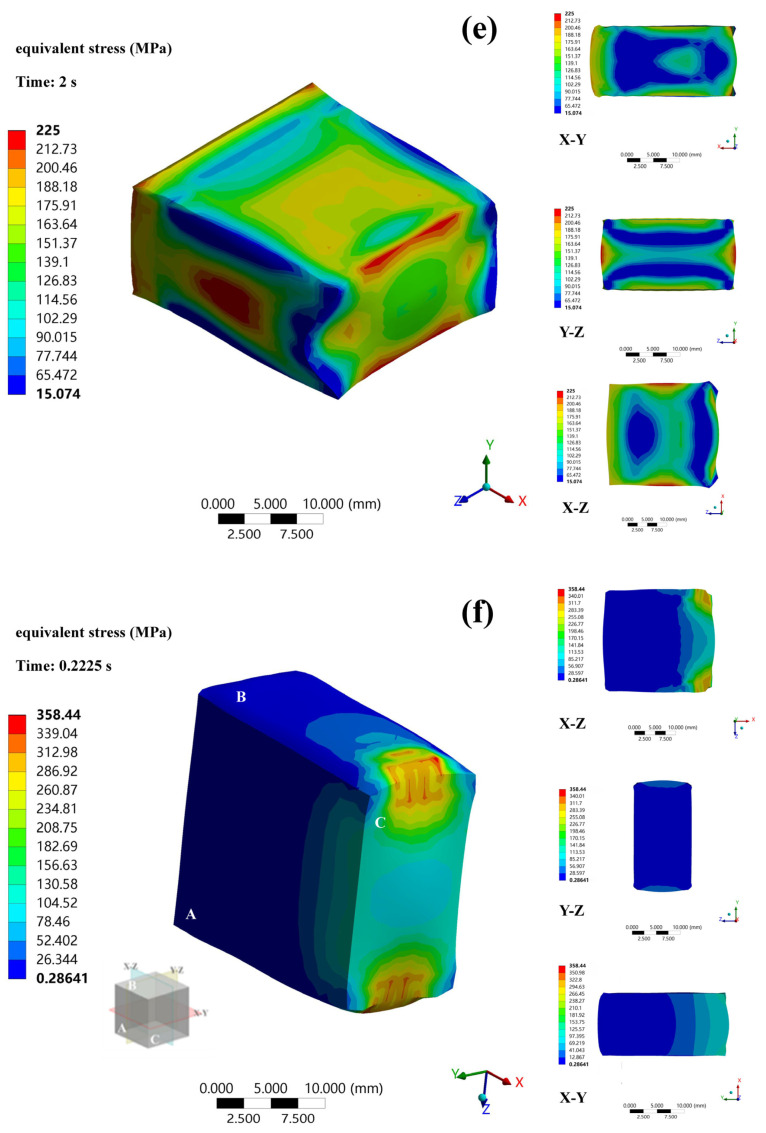

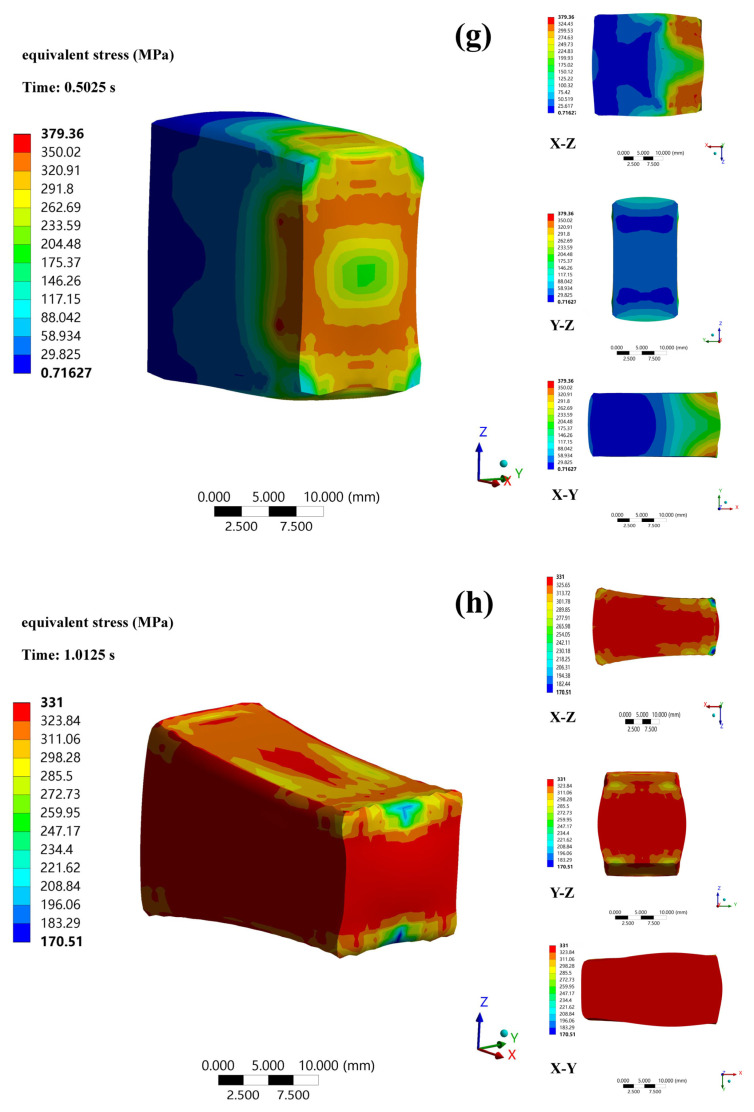

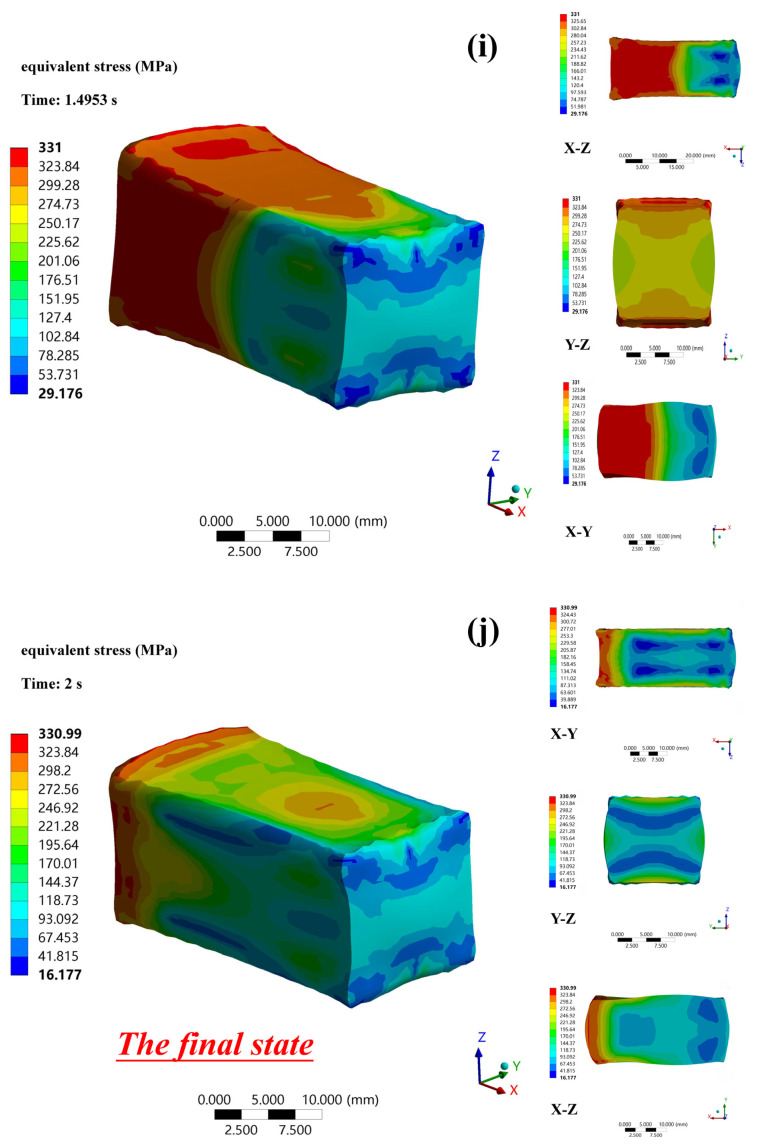
The equivalent stress distribution of MD rolled alloy. (**a**) Deformation of the rolled surface in 0.3225 s before turning over; (**b**) Deformation of the rolled surface in 0.56934 s before turning over; (**c**) Deformation of the rolled surface in 1.0065 s before turning over; (**d**) Deformation of the rolled surface in 1.5092 s before turning over; (**e**) Deformation of the rolled surface in 2 s before turning over; (**f**) Deformation of the rolled surface in 0.2225 s after turning over; (**g**) Deformation of the rolled surface in 0.5025 s after turning over; (**h**) Deformation of the rolled surface in 1.0125 s after turning over; (**i**) Deformation of the rolled surface in 1.4953 s after turning over; (**j**) Deformation of the rolled surface in 2 s after turning over.

The equivalent stress distribution of the alloy during deformation is depicted in Figure 7 and Figure 8. The Rigid Dynamics module of ANSYS Workbench 2021 was used to perform dynamic contact analysis using a second-order hexahedral grid with element type solid 186. A segment map was taken from the sample’s central position. Figure 7 shows the mesh distortions of the cross-sections of samples after conventional and MD rolling. The three-dimensional stress is primarily delivered to the contact surface during rolling in an ideal state, which is given by [29,30]:

(3) $$\sigma =\int_{S_{1}}^{\;}\left(\sigma_{0}\alpha_{s}+\tau_{x}\alpha_{sx}+\tau_{y}\alpha_{sy}\right)dS_{1}$$

where σ is the total compressive stress on the contact surface; $S_{1}$ is the area of the released surface; $\sigma_{0}$ is the unit rolling pressure; $\tau_{x}$ and $\tau_{y}$ are the longitudinal and transverse friction stresses of the contact surface, respectively; $\alpha_{sx}=-\frac{h_{x}^{\prime }}{2\sqrt{1+\frac{1}{4}h_{x}^{\prime 2}}}$, $h_{x}^{\prime }=\frac{\partial h}{\partial x}$; $\alpha_{sy}=-\frac{h_{y}^{\prime }}{2\sqrt{1+\frac{1}{4}h_{y}^{\prime 2}}}$, $\;h_{y}^{\prime }=\frac{\partial h}{\partial y}$; and *h* is the thickness of the sample.

After the current phase of the stress, input was finished. Structures such as DRX, dislocations, and vacancies were formed in the matrix. The non-uniform nucleation features caused variations in the DRX state of the grains, the quantity, the degree of plugging of dislocations, and the concentration of vacancies in various regions of the alloy [31,32]. The asynchrony caused differences in deformation resistance among these areas, which led to the simultaneous development of micron-sized grains with nanograins in the −196 °C rolled alloy. The A-side was always the ND surface during conventional rolling, so it bore the stress input. The excessive stress in the high-pressure area caused dynamic recovery (DRV), which induced the alloy to soften [33]. DRV occurred at the start of the crucial strain of DRX. MD rolling made the stress distribution of the alloy more uniform, and the occurrence of DRX was redistributed.

The relationship between the stress level F(*σ*) of various microscopic regions and the driving force $\sigma_{sat}$ for continuation of DRX in the hardened grains in this location is as follows [34]:

(4) $$F\left(\sigma \right)=\left\{\begin{array}{c} \sigma_{sat}^{n},\alpha \sigma_{sat}<0.8\\ \exp\left(\beta \sigma_{sat}\right),\alpha \sigma_{sat}>1.2\\ {\left[\sinh\left(\alpha \sigma_{sat}\right)\right]}^{n},for\;all\;\sigma_{sat}\;level \end{array}\right.$$

where α, n, and β are constants; and h is the work-hardening rate. Conventional rolling further inputs stress on the same contact surface. The $\sigma_{sat}$ values of the micro-regions cover a variety of ranges in the formula. During MD rolling, the grains in the block sample gradually acquire the strain, and the axial direction of its applied load is alternatively changed. In the microstructure, the original grains with unique orientations are gradually shattered and mosaicked. High-density dislocation and inter-dislocation solid contacts occur at crucial grain intersections. Proliferation and tangling of dislocations are more likely to transform into sub-grain barriers with low orientation variation in alloys with high stacking fault energy. The external force and grain boundary slip cause the sub-grain to develop into a unique fine recrystallization grain with a large-angle grain border. The load force axis transformation results in an increase in the number of fine recrystallization grains by increasing the number of junctions and intersection regions of various orientations [35]. The dislocations were not resolved because the Mg–16Li–1Zn–4Er alloy is a high stacking fault energy alloy. Therefore, through cross-slip and climbing operations, dislocations could quickly construct a substantial number of dislocation walls and substructures. However, more stored deformation energy was consumed in the MD rolling method, and the second-phase particles in the alloy and at grain boundaries generated significant pinning resistance. Therefore, the majority of the substructures had difficulty in completely evolving into DRX grains. The low-temperature deformation environment in this experiment further delayed grain growth and encouraged nanograin creation.

The contact surface switched from the A-side to the B-side for MD rolling. After initial rolling of the A-side’s surface, the B-side’s surface was the weakly stressed surface. The internal stress concentration of the microstructures on the B-side was low, allowing for higher stress input. The A-side only bore minor stress (*σ* A) caused by metal lateral flow when the B-side carried the primary stress input of rolling. Low stress lowers DRX levels. Due to the heterogeneity of the microstructures on the A-side, each microscopic area’s capacity to withstand stress input differed. A region in a low-stress state will absorb additional stress more easily than an area of high-stress concentration (F(*σ*) < *σ* A), and weak stress encourages DRX or DRV nucleation and a negligible amount of dislocation proliferation in the matrix. It is not, however, sufficient to achieve the necessary driving power for the significant junction area needed for DRV and dislocation slippage [36].

**Figure 9:**
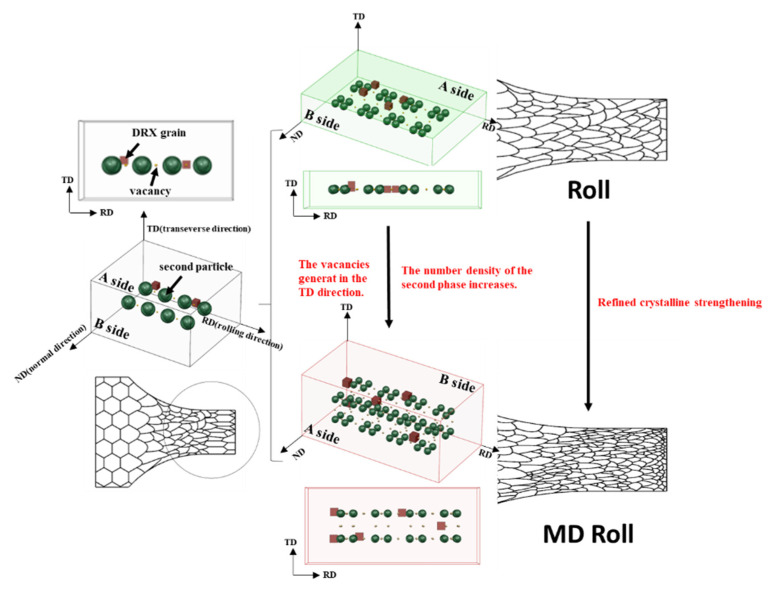
Schematic of distribution of the vacancies and broken second phase dispersion.

Additionally, because the alloy experienced compression from two directions, the primary second phase was completely broken and uniformly scattered, as seen in Figure 9. The contact ratio between the second-phase fragments and the matrix was increased. Additionally, the fragments were far apart, offering advantageous sites for nucleation of DRX [18,37,38]. This change had a significant effect on the increase in DRX nucleation rate compared to the rise in growth rate. The significant difference in microstructures between the 200 °C rolled, −196 °C rolled, 200 °C MD rolled, and −196 °C MD rolled alloys was encouraging. The small difference between the nucleation and growth rates, which were lower than similar parameters at 200 °C, may explain why the DRX grains of 25 °C MD rolled alloy were not greatly refined.

When the B-side was designated as the ND (normal direction) surface, the metal flow directions on the A-side and B-side surfaces were opposite to the previous deformation. No macro fractures were discovered in this material due to its extraordinary flexibility. The development of vacancies, on the other hand, was encouraged in the periphery of areas with varied deformation resistance. A sizable portion of vacancies existed as dislocations and DRX nucleation defects, which enhanced the alloy’s boosted strength [5]. Additionally, when compared to conventional rolling, MD rolling modified the stress distribution inside the matrix as well as the metal flow during the rolling process. It produced selective reinforcement for different microscopic regions and improved the matrix’s asymmetrical structure.

## 5. Conclusions

1. Rolling temperature and direction can both be used to maximize the alloy strengthening effect. The alloy gained maximum strength, increasing from 204 to 331 MPa, when MD rolled at −196 °C.
2. Multidirectional rolling broke the second phase of the alloy into finer sized and more uniformly distributed pieces than conventional rolling.
3. A large number of nanograins with a size of about 56 nm appeared in cryogenic multidirectional rolling. This facilitated the contribution of fine grain strengthening.
4. The low temperature effectively maintained the rate of DRX while considerably reducing the DRX grain growth rate, which was far lower than the nucleation rate. The grain refinement and dislocation effects were further optimized.
5. The results of the stress analysis showed that the modification of conventional rolling to MD rolling prevented the relaxation of high-stress areas while reinforcing weak-stress areas. A small amount of force applied in the microscopic area resulted in high-efficiency stress input.

## Data Availability

The data presented in this study are available on request from the corresponding author.
